# Draft Genome Sequences of Nine Cultivable Heterotrophic Proteobacteria Isolated from Phycosphere Microbiota of Toxic Alexandrium catenella LZT09

**DOI:** 10.1128/MRA.00281-20

**Published:** 2020-05-28

**Authors:** Xiao-Ling Zhang, Xi Yang, Shu-Jun Wang, Zhi-Wei Jiang, Zhang-Xian Xie, Lei Zhang, Jun Dai, Cheng-Qi Fan, Xiao-Qing Tian, Qiao Yang

**Affiliations:** a ABI Group, GPM Project, Shanghai, People’s Republic of China; b Innovation Laboratory of Systems Microbiology & Synthetic Biology, GAAS, Guangzhou, People’s Republic of China; c Laboratory of Systems Microbiology, Zhejiang Ocean University, Zhoushan, People’s Republic of China; d Jiangsu Key Laboratory of Marine Bioresources & Environment, Jiangsu Ocean University, Lianyungang, People’s Republic of China; e State Key Laboratory of Marine Environmental Science, Xiamen University, Xiamen, People’s Republic of China; f Shaanxi Key Laboratory of Agricultural & Environmental Microbiology, Northwest A&F University, Yangling, People’s Republic of China; g Key Laboratory of Fermentation Engineering of MOE, Hubei University of Technology, Wuhan, People’s Republic of China; h Laboratory of Marine Natural Products, East China Sea Fisheries Research Institute of CAFS, Shanghai, People’s Republic of China; University of Southern California

## Abstract

Microscopic interactions between phycosphere microbiota and host algae play crucial roles in aquatic ecosystems. Despite their significance, there is a scarcity of available genome sequences derived from the phycosphere microbiome. Here, we report the draft genome sequences of nine heterotrophic proteobacterial strains isolated from the toxic dinoflagellate Alexandrium catenella LZT09 during execution of our Phycosphere Microbiome Project. Further exploration of the genomic features of the alga-associated bacterial community will profoundly help in deeply deciphering the processes and mechanisms governing the host-microbe interactome within algal holobionts in the ocean.

## ANNOUNCEMENT

The phycosphere, the boundary of phytoplankton holobionts, is a unique microscopic niche for host-microbe interactions (1). Phycosphere microbiota inhabited within this distinctive microenvironment have been revealed in a number of phytoplankton assemblages and play crucial roles in aquatic environments (2). The dynamic interplay between host algae and the associated microbiota harbors cross-kingdom exchanges of nutrients, infochemicals, and gene transfer agents (2). However, there is a scarcity of available genome sequences of the phycosphere microbiota of bloom-triggering marine dinoflagellates. During the execution of the Phycosphere Microbiome Project, dozens of cultivable alga-associated bacteria were isolated from a globally distributed toxic dinoflagellate, Alexandrium catenella. Some strains have been identified as new members of the *Alphaproteobacteria* and *Gammaproteobacteria* (3–6). For deeply deciphering the mechanisms governing the host-microbe interactome of algal holobionts, draft genomic sequences of the nine bacterial strains are presented.

Bacterial strains of toxic *A. catenella* LZT09 were isolated according to our previously described protocol (5). For DNA extraction, the strains were cultured in marine 2216 broth at 25 to 28°C with shaking (150 rpm) for 36 to 48 h. DNA extraction and PCR amplification of bacterial 16S rRNA gene sequences of all strains were performed according to the methods described previously (5). The strains were identified taxonomically based on the comparison of 16S rRNA gene sequence similarity using the online EzBioCloud database (http://www.ezbiocloud.net/eztaxon) (7). Genomic DNA was extracted with DNeasy UltraClean DNA kits according to the instructions from Qiagen (Maryland, USA). The Illumina 2 × 250-bp paired-end library was prepared using a TruSeq DNA sample prep kit from Illumina (Massachusetts, USA) according to the manufacturer’s instructions and then sequenced using an Illumina HiSeq 4000 system. Trimmomatic v0.36 with default settings was used for trimming and quality filtering (8). FastQC v0.11.2 (http://www.bioinformatics.babraham.ac.uk/projects/fastqc) was used to assess the read quality. The genome assembly for strains of LZ-5^T^, LZ-14, and LZ-16-2^T^ was performed using SOAPdenovo v2.04 (9), and the other six strains were done with SPAdes v3.5.0 (10). Gene prediction and genomic annotation were performed using NCBI PGAP v1.2.1 (11). Default parameters were used for all software unless otherwise specified. All of the genomes sequenced exceeded 260× coverage, and the characteristics of the nine obtained assemblies are described in Table 1. The availability of genome data for the phycosphere microbiome of toxic *A. catenella* will offer valuable clues for ongoing comparative studies on alga-bacterium interactions.

**TABLE 1:** Summarized genomic features of nine alga-associated heterotrophic proteobacteria isolated from the cultivable phycosphere microbiota of toxic *Alexandrium catenella* LZT09

Strain name	Genome length (bp)	G+C (mol%)	No. of genes	No. of reads	Coverage (×)	No. of contigs	*N*_50_ (bp)	Assembler	SRA accession no.	GenBank accession no.	No. of tRNAs	No. of rRNAs	No. of ncRNAs^*a*^
*Limnobacter* sp. LZ-4	3,488,419	52.5	3,298	8,534,482	422	17	452,966	SPAdes v3.5.0	SRR11279520	SWKN00000000	3	39	4
Saccharospirillum alexandrii LZ-5^T^	4,712,158	56.4	4,222	10,915,266	326	29	1,318,875	SOAPdenovo v2.04	SRR11267958	RCIP00000000	3	53	4
*Marinobacter* sp. LZ-8	4,337,754	57.4	3,998	9,344,736	312	20	882,613	SPAdes v3.5.0	SRR11300914	SWKM00000000	4	49	4
*Ponticoccus* sp. LZ-14	4,615,538	63.3	4,576	12,561,168	408	111	1,271,123	SOAPdenovo v2.04	SRR11268417	RBVZ00000000	3	40	3
*Marivita* sp. LZ-15-2	4,829,700	59.2	4,745	9,197,832	263	65	380,371	SPAdes v3.5.0	SRR11267608	SWKO00000000	6	42	3
*Maricaulis* sp. LZ-16-1	3,348,699	63.6	3,210	10,744,348	461	12	558,629	SPAdes v3.5.0	SRR11266721	SWKP00000000	3	43	4
Haliea alexandrii LZ-16-2^T^	3,961,381	61.3	3,606	10,318,956	367	12	905,050	SOAPdenovo v2.04	SRR11278742	RFLW00000000	3	41	4
*Maritimibacter* sp. LZ-17	4,309,600	64.3	4,269	10,762,194	375	21	937,164	SPAdes v3.5.0	SRR11266783	SWKQ00000000	3	41	3
*Mameliella* sp. LZ-28	5,663,795	64.9	5,548	14,028,028	372	38	326,544	SPAdes v3.5.0	SRR11285606	JAANYX000000000	8	50	3

a ncRNAs, noncoding RNAs.

### Data availability.

The genome sequences of the nine bacterial strains were deposited in DDBJ/ENA/GenBank. Detailed information is listed in Table 1.
